# An emerging form of public engagement with science: Ask Me Anything (AMA) sessions on Reddit r/science

**DOI:** 10.1371/journal.pone.0216789

**Published:** 2019-05-15

**Authors:** Noriko Hara, Jessica Abbazio, Kathryn Perkins

**Affiliations:** 1 Information & Library Science, School of Informatics, Computing & Engineering, Indiana University, Bloomington, Indiana, United States of America; 2 University of Minnesota Libraries, University of Minnesota Twin Cities, Minneapolis, Minnesota, United States of America; Universidade do Porto Instituto de Biologia Molecular e Celular, PORTUGAL

## Abstract

Originally, online public engagement with science tended to be one directional—from experts to the general population via news media. Such an arrangement allowed for little to no direct interaction between the public and scientists. However, the emergence of social media has opened the door to meaningful engagement between scientists and the general public. The current study examines scientists’ perspectives on the interactions between laypeople and scientists by asking questions and sharing information on social media platforms, specifically, through Ask Me Anything (AMA) sessions on Reddit’s “Science” subreddit (r/science). By analyzing the content of six different r/science AMAs and surveying scientists who participated as r/science AMA hosts, our research attempts to gain a richer understanding of direct communication between scientists and lay audiences online. We had three main questions: (1) who are the participant scientists hosting r/science AMAs, (2) what are their experiences like as hosts, and (3) what type of discussions do they have on this platform? Survey results suggested that these scientists recognize the promising interactive nature of Reddit and are interested in continuing to use this platform as a tool for public engagement. Survey respondents generally had positive experiences as AMA hosts, but further research is needed to examine negative experiences. Overall, this study has significant implications for how scientists can engage public audiences online and more effectively communicate scientific findings to the general populace.

## Introduction

Engagement with the general public is an important scientific responsibility. By effectively communicating scientific knowledge, researchers provide citizens with the facts needed to make informed decisions, encourage the public to value and be more interested in science in general, and, hopefully, create a climate where there is greater public support for funding scientific research [1]. Originally, communicating about science with the general public was largely one-directional—from experts to the general public via news media. Recently, the term Public Engagement with Science (PES) has become more widely used to emphasize active engagement with the public through public participation [2–3]. For example, Einsiedel [4] proposed three categories of public participation: policy making, dialogue, and knowledge production. Policy making refers to citizen participation in policy-influencing activities, such as panels, polls, and juries [5]. Dialogue includes engagement in science cafés, festivals, and science exhibits that encourage conversation between citizens and scientists [6]. Knowledge production has increased in more recent years through citizen science projects, such as Galaxy Zoo [7]; crowdsourcing, such as Patients Like Me [8]; and collaboration with scientists, such as the study of the French Muscular Dystrophy Association (AFM) [9]. Lately, access to the Internet has changed how the public engages with and participates in science and technology research [4]. In this paper, we specifically address the second type of public participation, i.e., dialogue.

While public engagement with science (PES) activities traditionally inhabit physical environments, such as museum exhibits [10] and science festivals [11], this paper focuses on PES in an online environment. As more people than ever before access scientific information on the Internet [12], there is an increasing need to examine online PES activities so that scientists can continue to develop effective communication strategies to reach the public.

Prior studies of online PES examined scientific communication within the context of the one-directional model, investigating the roles of traditional mediators (journalists, healthcare professionals, government organizations, etc.) in facilitating the transfer of scientific knowledge from scientists to the general public (e.g., [4][13]). Even though engagement in robust dialogue with the general public has been encouraged (e.g., [14]), dissemination of knowledge continues to be the primary focus of scientists’ interactions with the public [15]. However, this waterfall model of communication is being challenged. Brossard [16] notes that the role of lay participation facilitated by online environments is changing the nature of science communication. Today, new challenges for effectively communicating scientific knowledge are emerging; communication is no longer linear. The prevalence of online communication, especially on social media platforms, is creating both opportunities and challenges for scientists seeking to effectively communicate scientific knowledge to the general public. Opportunities include the ability for scientists to reach out to larger populations directly, without needing to leave their physical offices. Challenges include time constraints, unpleasant interactions, and widespread misinformation.

Online Public Engagement with Science using social media has the potential to be more bi-directional. Research about online science communication, and more specifically the use of social media for science communication, has flourished in recent years. Lately, the journal *Science Communication* published a special issue entitled “Public science in a wired world: How online media are shaping science communication” [17]. Contributors critically examined a variety of social media uses for science communication. For example, Su, et al. [18] analyzed Twitter use during a science festival called NanoDay. They found that tweets related to this event were largely informational (one-way), although there were some tweets related to soliciting participation (e.g., sharing photos of the event) and volunteer opportunities. Vraga and Bode [19] noted the importance of correcting misinformation online in an experimental study involving the Zika virus. They used Twitter feeds that were constructed specifically for this study and found that corrections made by authoritative organizations, such as the Centers for Disease Control (CDC), were especially effective among student participants in the experiment.

Yet even in an age where social media plays a significant communicative role, online PES remains primarily one-way and focuses on the dissemination of knowledge, not on the cultivation of engaging dialogues. For example, Kahle, et al. [20] analyzed public engagement with different types of social media using controlled content on the social media platforms of the European Organization for Nuclear Research (CERN). They posted 48 different topics on five of CERN’s social media platforms, including two Twitter accounts (in English and French), Facebook, Google+, and Instagram, over eight weeks in 2014. Not surprisingly, they found that images that inspire amazement (e.g., CERN dishwasher for circuit boards) received more likes, click-throughs, and shares. In another study, Collins, Shiffman, and Rock [21] conducted a survey of over 500 scientists from various disciplines and reported that nearly all respondents widely used social media in their work lives, using platforms such as Facebook and Twitter; however, when describing their colleagues’ habits, scientists reported that the use of social media to engage the public in a discussion of their research was not yet widespread. Furthermore, their social media uses tended to be limited to more of what Peters, et al. [22] called a “self-presentation of science.” This means that scientists who utilize social media tend to make announcements about their work rather than engage in dialogues with the general public. This type of social media use can be educational but remains one-way and does not necessarily encourage public participation [18][23].

Studies that focus on understanding scientists’ perspectives reveal this tendency of online PES to center on top-down knowledge dissemination. For example, Dudo and Besley [1] surveyed 390 members of the American Association for the Advancement of Science to examine their objectives when they reach out to the general public online and how these objectives shape scientists’ communication strategies. They found that defending science was the top priority. In addition, they discovered that scientists’ preconceived notions of their audience significantly affected how they communicated. Another study by Jensen and Holliman [15] asked about science communicators’ (including scientists’) activities and experiences while engaging with the general public; respondents said that they focused primarily on addressing the knowledge deficit—the so-called Deficit Model [24] of science communication. The Deficit Model assumes that “ignorance is the basis of a lack of societal support for various issues in science and technology” ([25] p. 401).

Members of the general public also engage with science online, outside of social media. For example, citizen science projects on classifying astronomic data, such as Galaxy Zoo [26] and Wikipedia editing [27], involve two-way interactions. These platforms make it easier than ever to invite laypeople to participate in this type of online PES—whether it be sharing parental tips for combatting head lice [28], discussing autism [29], or sharing scientific findings [30]. In these settings, laypersons (i.e., the intended audience) are not simply passive recipients but active contributors in the collaborative construction of science on go-to social media platforms.

Another notable example of two-way online communication is Reddit. Even though relatively few scientists use Reddit, according to a survey by Collins, Shiffman, and Rock [21], some reports indicate that it has great potential to connect scientists directly with the general public, especially with those who are interested in science (e.g., [31–32]) and health-related topics [33]. In fact, Reddit has a dedicated sub-category (known as a “subreddit” on its site) called “Science” (reddit.com/r/science), where people discuss a variety of scientific topics. Owens [32] called Reddit’s science-focused subreddit “the world’s largest 2-way dialogue between scientists and the public.” Dudo [34] also lists the “Science” subreddit as a promising area of research in terms of examining the communication between scientists and the general public online. The question-and-answer format of the site and its participatory nature (anyone can contribute) allow this platform to provide emerging forms of science communication that are more interactive.

### Research questions

Previous research on Reddit’s use for online PES in science is essentially descriptive. As such, we were interested in fully exploring, from the scientists’ perspective, this popular two-way forum for interactions between scientists and the general public, specifically as it occurs in the “Science” subreddit. This type of two-way science communication significantly differs from traditional scientists-and-lay-people-interaction spaces, like science cafés and science exhibits [4]. Furthermore, online PES is of interest to communities of scientists [1]. With this in mind, we conducted an exploratory study in order to unbox this emerging form of science communication by asking the following research questions:

- Research Question (RQ)1: What kind of demographic characteristics do the scientists participating in “Science” subreddit AMAs have?
- RQ2: What was the experience like to host an AMA in the “Science” subreddit?
- RQ3: What type of discussions did “Science” subreddit AMA participants engage in?
  - RQ3a. Do questions receive answers?
  - RQ3b. What are posters’ intentions?
  - RQ3c. What kind of content features appear?
  - RQ3d. Who is posting comments?
  - RQ3e. What kind of responses do posts receive?

We chose to focus on the “Science” subreddit (hereafter referred to as r/science) to study an emerging form of public engagement in science. r/science was created in October 2006 and has attracted approximately 20 million subscribers as of January 2019. Reddit sponsors sessions called “Ask Me Anything” (AMAs), which invite experts to answer questions that Reddit users ask. Until May 2018, when the subreddit r/science ceased hosting AMA sessions, r/science presented up to five AMAs a week, but not more than one per day to avoid overlaps. Past r/science AMA hosts included leading scientists in the fields of genetics, climate science, and space exploration, as well as science celebrities like Stephen Hawking. r/science began hosting AMAs in January 2014 with an initiative by the chemist Nathan Allen who envisioned discussions between scientists and the public on Reddit (https://en.wikipedia.org/wiki//r/science). r/science AMAs gained popularity among scientists to the extent that some professional organizations, such as the American Chemical Society and American Association for the Advancement of Science, as well as academic journals such as PLOS ONE, sponsored r/science AMA sessions. Scientists went through a verification process so that users could be assured that the individuals hosting AMAs were actually accredited scientists. Participants also had the opportunity to verify their expertise. For example, participants could receive “flairs” that denoted they were a verified scientist, engineer, student, etc. Flairs were a way for Reddit users to prove that they were providing educated opinions on a topic and had the credentials to support their opinions. After the r/science moderators verified a user’s credentials, the user’s account was assigned descriptors that informed others of that user’s level of education in a specific discipline. Examples of tags included: "biology,” “neuroscience,” “environment,” and “animal science.” See more information at: https://www.reddit.com/r/science/wiki/flair. Unfortunately, the moderators of r/science decided to discontinue AMAs on their subreddit in May 2018 after changes were made to the Reddit “upvote” algorithm, which caused participation in r/science AMAs to drop precipitously. However, similar outlets still exist, such as the r/IAmA subreddit and the generic r/AMA subreddit. In the former, there is still a flair mechanism that allows hosts to tag a specific IAmA as science-related (as opposed to celebrity, politics, etc.).

## Material and methods

### Ethics statement

Indiana University’s Institutional Review Board (protocol # 1703890480) approved the study. An informed consent form was presented to all participants, and written consent was received when participants agreed to respond to the survey.

### Data collection

To gain an overview of who the scientist AMA hosts were and what they discussed with the general public, we used a mixed method approach, employing two data collection methods: survey and content analysis.

### Survey

A survey that consisted of 26 questions was distributed using the online Qualtrics platform (see S1 File). The questions included demographic information, such as age, gender, race, and discipline. They also addressed the experience of hosting an AMA, favorite types of questions, lessons learned, and the reasons for agreeing to host. In order to gain access to the scientists who hosted AMAs, we harvested 315 scientists’ names from the r/science subreddit and identified their contact and demographic information in June 2017 and distributed the survey via email later that month. Among them, 73 responded and 70 completed the survey (a 22.2% completion rate). Because survey research suggests that reminders improve online survey response rates [35], we sent two reminders in the first week and then another, two weeks after the initial invitation email.

### Content analysis

We selected six AMAs for detailed content analysis (see Table 1). Selection criteria were session timing, length of discussion, and discipline. First, the selected AMAs were chosen because they all occurred during the four months prior to the distribution of the survey. Second, to facilitate our manual coding, we selected sessions with 200 to 300 responses. Finally, we chose four AMAs that represented science disciplines that were covered relatively frequently by r/science AMAs (astronomy, biology, chemistry, and geology). Moreover, we noted that the most common r/science AMAs focused on medicine and environmental science, and not on more traditional science disciplines such as physics (see S1 Table). As such, we selected two additional r/science AMAs in medicine and environmental science.

**Table 1:** Overview of AMAs coded for content analysis.

	AMA #1	AMA #2	AMA #3	AMA #4	AMA #5	AMA #6
Discipline	Astronomy	Biology	Chemistry	Env. Sci.	Geology	Medicine
Topic	NASA	Bird Genetics	Biomedical nanomaterials	Climate Change	Snow and Ice	Wound Dressing
Total # of posts	227	203	236	248	198	251
# of hosts	5	2	1	2	1	6

Our codebook originated from work by Jeng, et al. [36], who examined question and answer (Q&A) posts on an academic social networking site called Research Gate. Because AMA participants are also engaged in Q&As, we adapted five categories from their original codebook with major revisions: poster’s intentions (PI), content features (CF), poster’s identity (PID), comment status (CS), and answer status (AS). Poster’s intentions considers the goals and expectations of the hosts and participants. Content features examines the substance of posts. Poster’s identity determines who the author is for a specific post—either host, participant with a flair, or participant without a flair. Finally, answer status and comment status specify whether questions were answered by the host and/or were commented on by other participants.

To create the codebook used for this study, we examined the content of a single AMA (Geology) and added new codes or modified the definitions of codes that appeared in the list developed by Jeng, et al. [36] in order to more accurately describe the content present in the AMA. After coding an initial AMA, we modified the original codebook (see S2 Table for the codebook). Whenever a question or uncertainty about how to apply a code arose, we modified or clarified the definition of the code to better describe the content of the AMAs, which allowed for codes that were easier to apply and more universally descriptive of AMA content. Sometimes, we added codes and then deleted them from the codebook when they did not apply to the AMA content in expected ways. For example, one draft of the codebook included a code that applied to posts that “made references,” which could possibly have applied to references to resources, publications, theories, or scholars. This code proved to be problematic when we considered that “references” could also apply to mentions of specific historical or current events or vague allusions to unnamed sources (e.g., “I’ve been watching videos and reading about tree wells” or “there are many stories of people falling into crevasses”). This question was further complicated when we considered whether “making references” should apply only to pieces of information that were general knowledge or to ideas that required specialized knowledge about a scientific field to understand (e.g., deciding if a reference to “pressure melting” or a “Heinrich event,” concepts that would be common knowledge to geologists who specialize in glaciers but not to a layperson, should count as “making a reference” in the context of coding). Lastly, we modified the codebook in instances that necessitated further refinement of the codes presented by Jeng, et al. [36] or earlier drafts of our own codes. For example, the Jeng, et al. codebook included simple distinctions for posts that include a question (i.e., QI1. Seeking information and QI2. Seeking discussion). The authors carried that concept over into the present study (PI1. Seeking information and PI2. Seeking discussion), but allowed for further refinement of the coding of question posts by adding a category of content feature code (“Making an inquiry”) and providing granularity that illustrated not only that the poster’s intention was to ask for information or spark discussion, but to indicate that the post contained a question (rather than a statement) and to highlight when and how the question appeared within the wider context of the discussion (CF6a. Making an inquiry–initial question and CF6b. Making an inquiry–embedded question).

The revised codebook included: poster’s intentions (PI), answer status (AS), comment status (CS), poster’s identity (PID), and content features (CF). Answer status and comment status assess whether a post was answered (if it contained a question) or was commented upon, in order to evaluate which posts generated answers and follow-up discussion. The poster’s identity was used to examine who was contributing and what types of contributions they were making. In order to test these codes, two of the authors coded individual AMAs separately and compared the results to find discrepancies in their approaches to coding. The authors discussed the thought processes behind their coding and whether these difference in interpretation resulted from issues of clarity concerning the definition of the code in question. In coding the different AMAs, we found new concepts that had not arisen in AMAs coded previously, events that necessitated reevaluation of code definitions so that they could be used more universally. Whenever disagreements arose between the two authors, the codebook was revised to address the issue. We tested new versions of code definitions by separately coding another AMA and comparing the results.

Some 20% of the postings coded were analyzed by two of the authors; inter-coder reliability ranged between 0.66 and 1.0 calculated by Cohen’s Kappa. According to McHugh [37], inter-coder reliability rates between 0.61–0.80 are considered substantial and between 0.81–1.0 are almost perfect agreement. From this analysis, the codebook appeared to be robust.

Due to differences in the total number of posts for the six selected AMAs, we calculated percentages for the code results in order to compare AMAs. When a single code was presented, the percentage was calculated by using the total number of codes for that particular category (e.g., poster’s intention). When a combination of codes was presented, the percentage was calculated by using the total number of posts for that particular AMA (e.g., AMA #1).

## Results

### Who are r/science AMA hosts?

We harvested contact information from 315 scientists who hosted AMAs in r/science that occurred between October 20, 2016 and June 27, 2017. First, we analyzed these scientists to identify who they were with regard to demographics (RQ1). The data (see S2 Table) revealed that the ratio between female and male was approximately two to three, the majority of hosts (70.5%) had a terminal degree, and most of those who had terminal degrees had a Ph.D. (91.9%). The majority of AMA hosts (80%) worked in U.S. institutions. Overall, hosts came from 18 different countries primarily concentrated in North America and Europe. Finally, these AMA hosts represented 19 different areas of science ranging from more recurrent categories, such as Medicine (15.2%) and Biology (14.6%), to less frequently hosted categories, such as Animal Science (1.0%) and Paleontology (0.6%).

Of the 315 scientists who were contacted, 70 completed the survey. Their demographic information is shown in the S3 Table. Some questions (e.g., those about race or how the scientists came to be involved with hosting AMAs) allowed multiple answers. Percentages were calculated based on the total cumulative number for each category.

Survey respondents’ gender was quite balanced (51.4% of those who responded were men and 45.7% were women) when compared with both Reddit’s general user profile (67% men; 33% women) and those who typically host an AMA in r/science (see S2 Table). Reddit user demographics came from Pew Internet Research: http://www.journalism.org/2016/02/25/reddit-news-users-more-likely-to-be-male-young-and-digital-in-their-news-preferences/. It was not surprising that the survey respondents were highly educated; 74.3% had doctoral degrees and 14.3% had master’s degrees, although the percentage of Ph.D. degrees was lower than the profiles of AMA hosts found in S2 Table. It was also not surprising that they were relatively young: 42.9% of the respondents were in their 30s and 18.6% were in their 20s. This is consistent with Reddit’s user profile—93% of users are younger than 49 (64% of users are aged between 18 and 29 and 29% are aged between 30 and 49). The survey respondents were predominantly white (90.4%) and most of them (71.4%) worked in universities.

When it came to deciding to participate in an AMA, encouragement from colleagues was key (32.5%). At the same time, professional associations also played a significant role in encouraging scientists to host AMAs (19.3%). Finally, when asked about the duration of their involvement in r/science, most of the survey respondents noted that they were new to this subreddit. Approximately 70% of the respondents began reading r/science within the previous year, and 80% of them only started participating actively in r/science less than a year before completeing our survey. A cross-tabulation showed no specific patterns for those who rated their experience as “neutral,” partially because there were so few.

### What was the experience like to host an AMA in the “Science” subreddit?

We then examined the experiences that r/science AMA hosts had (RQ2). The majority of the survey respondents were overwhelmingly positive about their experience hosting an AMA. Approximately 95% of the respondents answered either positive or very positive to the question: how positive or negative was your experience hosting an AMA? Only 5% of the respondents selected neutral to the same question, and none answered either negative or very negative. This result was unexpected; we anticipated that at least some would have had negative experiences. If any r/science AMA hosts had bad experiences, they did not respond to our survey.

The rest of the questions were open-ended. When asked about the most enjoyable questions from r/science participants, the survey respondents’ answers were generally about research-focused questions, “intelligent” questions, and questions that had research implications. One of the respondents answered:

*The questions that I enjoyed the most were the "life at sea questions," or the "How do you do xy&z at sea?" I also really enjoyed questions from younger people interested in what career paths we had taken to get work in our current fields*.

When asked about the questions they found least enjoyable, those who responded answered that they did not appreciate unpleasant questions (i.e., questions which were off-topic, repeated, and/or antagonistic), personal attacks, and leading questions. Some comments from respondents included: “*A few troll like questions did get through*…,” “*Conspiracy theory*,” and “*Ones about funding the space program*, *or ones about how NASA is covering up stuff*.”

When asked about the most enjoyable aspects of their experience hosting, two main themes came up: interacting with the public and collaborating with other scientists hosting the AMA. For example, one of the respondents said:

*I did not have any previous experience of chatting in public and was a bit nervous about it, but I found that once it got started it was great fun to try to answer as many questions as succinctly, accurately, and pedagogically as possible*.

This respondent had never interacted with the public in the role of a science communicator, yet they had a positive experience hosting an r/science AMA. According to previous research (e.g., [38]), scientists are not necessarily excited about interacting with the public; some consider science communication an unwelcome obligation. However, the enthusiastic response above suggests that the respondent will likely promote a platform like the r/science AMA for activities related to online PES in the future.

The positive response to collaboration with other scientists was somewhat unexpected. We had assumed that scientists coordinated their answers if there were multiple hosts for an r/science AMA, but this collaborative aspect of answering questions was one of the positive aspects that was mentioned frequently. One respondent who participated in a multi-host AMA commented: “*Sitting in a room with my colleagues*, *discussing which questions each of us would answer*, *was a really fun*, *bonding*, *outreach experience*.” Scientists often collaborate on scientific projects [39], but science communication tends to be accomplished individually. Emphasizing the collaborative aspects of online PES may encourage more scientists to engage with the general public.

We also asked about scientists’ least enjoyable experience while hosting an AMA. The answers varied, but, in summary, the following four issues were raised: technical problems, time limitations, time differences (for example, between the US and UK), and not being able to answer all of the questions. The first issue is inevitable when dealing with technologies. The challenges can appear daunting but can be expected to diminish as these technologies become more commonplace. The second issue (that of time) relates to the fourth issue. Effective time management skills are helpful when hosting Reddit AMAs, which are intense and occur during a short period of time. The third issue relates to synchronous online interactions. When dealing with global collaboration, the problem of time zones frequently arises [40]. This is certainly an unavoidable issue, and hosts and participants need to compromise.

However, these drawbacks must not have been major issues because the majority of survey participants (93.0%) responded that they would either be likely or extremely likely to host an AMA session again. The following description gives a succinct account of what it is like to host an AMA in r/science: “*This was a very fun experience for all of the scientists on board—we each took a break from heavy science and had the opportunity to speak on a very basic level with a general audience about our science*. *We had a great time*!” This quote is consistent with a finding that interactions with the general public could help scientists become more reflective about their own research [41].

These survey responses helped us understand some of the scientists’ perspectives on hosting r/science AMAs. Next, we analyzed the content of those interactions that occurred in r/science AMAs.

### What’s happening in r/science AMAs?

During the period of investigation, the maximum number of posts per session was 2,469, the minimum number of posts per session was 17, and the average number of posts was 275. The average number of participants per session was 113. No scientists repeated AMA hosting during this period. By using content analysis, we investigated the types of discussions in which r/science AMA participants engaged (RQ3).

#### Answering questions

When we analyzed the code, Answer Status (RQ3a), we found that the number of questions posed and the percentage of which were answered during an AMA varied (see Table 2). The host, or sets of hosts in the case of four of the AMAs, responded to about 50% of participants’ questions. Two sets of hosts responded to more than 70% of questions, but one replied to less than 20%. Considering the two AMAs with higher response rates, we observed that the responses by the two hosts in the biology AMA were very succinct and to the point. It appeared that they aimed to respond to as many questions as possible by keeping their answers brief. The Medicine AMA had six researchers answering questions. They seemed to be able to divide the labor of answering questions among themselves effectively—another key factor in answering more questions successfully. The AMA with the lowest response rate was the Chemistry AMA, which focused on the topic of nanomaterials and only had one host. Although the answer rate was lower, the detailed responses this researcher provided were impressive. The answers were generally a paragraph or more, unlike other AMAs in which responses could be as short as a sentence. The answers in the Chemistry AMA also included specific scientific references; we did not observe any links (i.e., URLs) in the answers provided by the host of this AMA. It appeared that the host preferred to offer thorough answers in as clear terms as possible and that links to websites were not likely to satisfy this host’s standards. Here, the researcher seemed to emphasize quality (i.e., providing detailed answers) over quantity.

**Table 2:** Number of questions and response rates.

	AMA #1 Astronomy	AMA #2 Biology	AMA #3 Chemistry	AMA #4 Env. Sci.	AMA #5 Geology	AMA #6 Medicine
Question Response Rate	52 (49.5%)	59 (70.2%)	30 (18.4%)	60 (57.1%)	47 (45.6%)	78 (74.3%)

Note: Percentages were calculated based on the number of posts coded AS1 (Answered) divided by the total number of posts coded for AS1 (Answered) and AS2 (Not answered).

#### Intention to answer questions

Not all of the posts in an AMA were answering questions. One of the code categories that we used was to identify poster’s intentions (see the S3 Table for the codebook), which included making a comment that did not directly answer a question or asking a follow-up question (RQ3b). Table 2 includes the numbers and percentages of posts that were coded PI5, meaning the poster—whether they were a host or a participant—intended to answer a question. The trends for the six AMAs are the same as the hosts’ response rates reported in the previous section (see S1 Fig). In other words, AMA #2 (Biology) and AMA #6 (Medicine), as well as AMA #4 (Environmental Science), have higher percentages than others, and AMA #3 (Chemistry) has a lower percentage.

**Fig 1:**
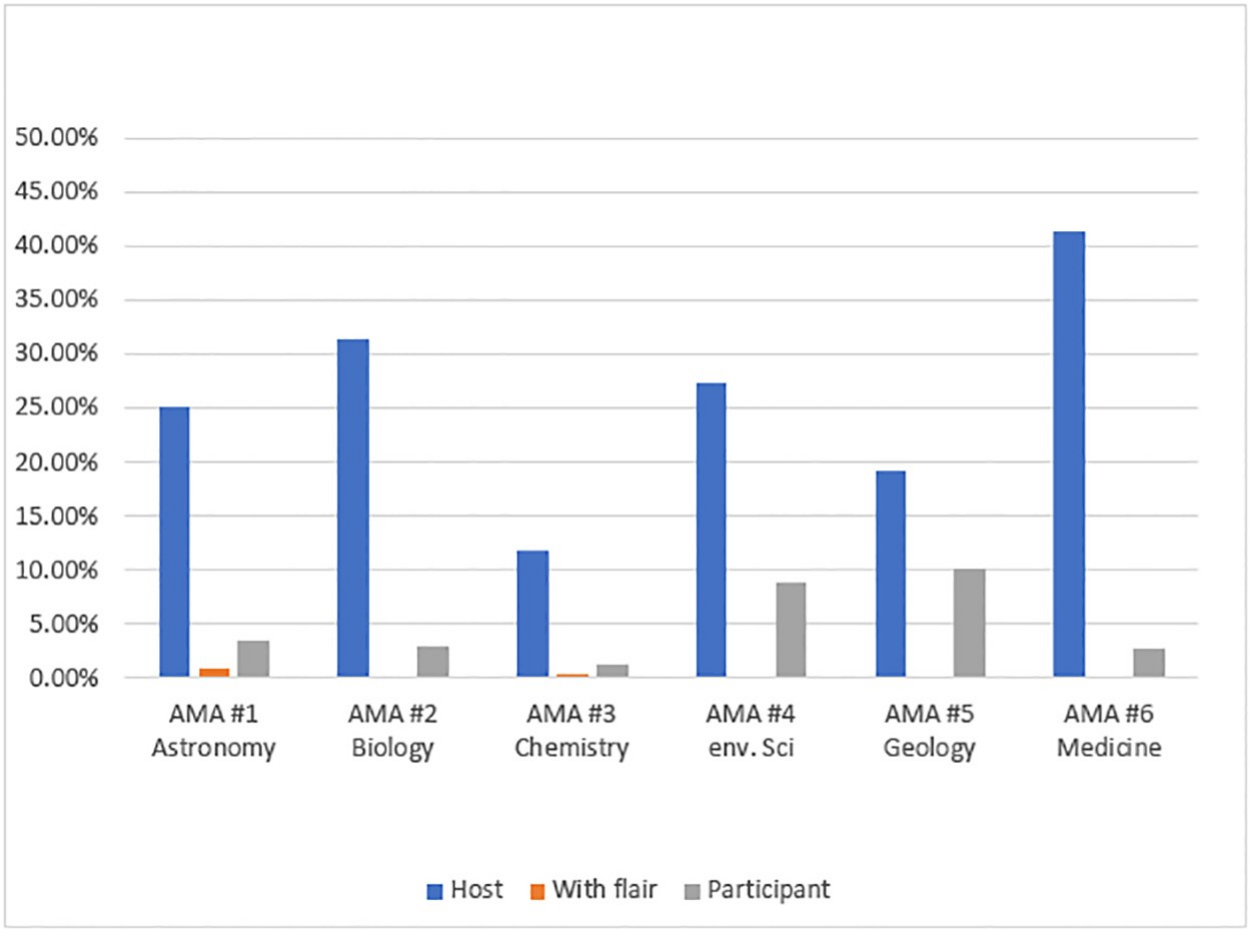
Percentages of posts by AMA hosts and participants containing direct answers to questions (PI5+ PID1/2/3). Note: Percentages were calculated by the number of posts coded as PI5 + PID1/PID2/PID3 divided by the total number of posts.

We further examined the numbers and percentages of posts by different types of participants that intended to answer questions (Fig 1 and S4 Table). The code combination of PI5 (poster’s intention to answer a question) and user types—PID1/PID2/PID3 (host/s, participants with a Reddit credential flair, or participants without a flair)—showed that most of the posts answering a question were written by the AMA hosts, especially in AMA #6 (Medicine). At the same time, almost 10% of posts coded “intended to answer questions” were by participants without a flair in AMA #4 (Environmental Science) and AMA #5 (Geology). The number of answers provided by participants with a flair were less. Even though posts answering questions by participants with a flair were limited, these answers were insightful; we speculate that the audience would value these answers more than answers provided by participants without a flair.

#### Types of discussions

**Fig 2:**
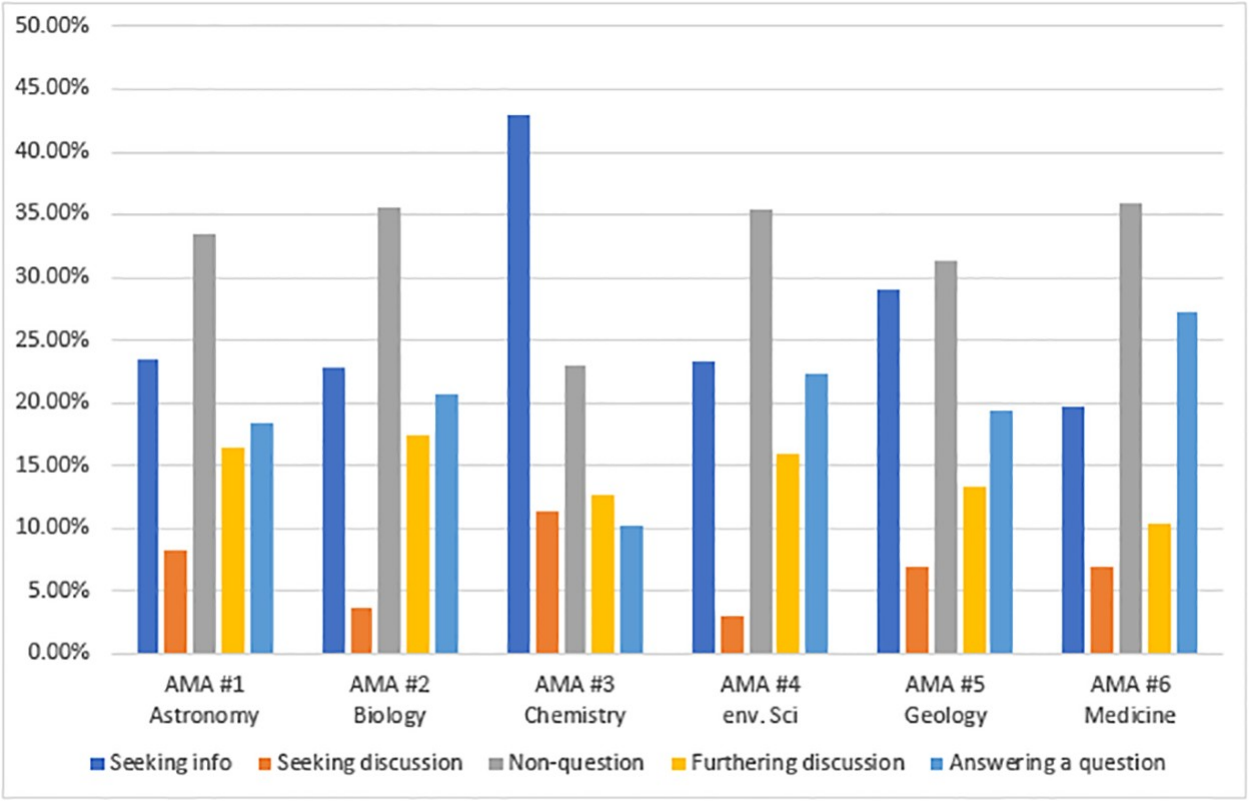
Poster’s intention. Note: Percentages were calculated by the number of posts for each code divided by the total number of all the posts coded for PI (i.e., Total #s in the table).

Fig 2 and S5 Table show the frequency of codes for poster’s intentions (RQ3b). In terms of poster’s intentions, seeking information and answering a question occupied approximately half the posts for each AMA (41.8%, 43.4%, 53.0%, 45.7%, 48.3%, 46.9% for each AMA respectively). This makes sense in that questions and answers are the purpose of the Ask Me Anything forums on Reddit and the participants evidently focused on asking and answering questions. However, there were some variations. For example, AMA #3 had more posts seeking information than average, and AMA #6 had more posts answering questions than average. It is possible that the topic for AMA #3 (nanomaterials) was considered “basic science” based on fundamental research and that people were more likely to ask specific questions, whereas the topic for AMA #6 (wound dressing) was considered “applied science” and was therefore more relevant to daily life. As such, hosts and participants in AMA #6 were more inclined to contribute by answering questions rather than simply seeking information. In addition, AMA #6 had more hosts (six) than the other AMAs included in this analysis.

**Fig 3:**
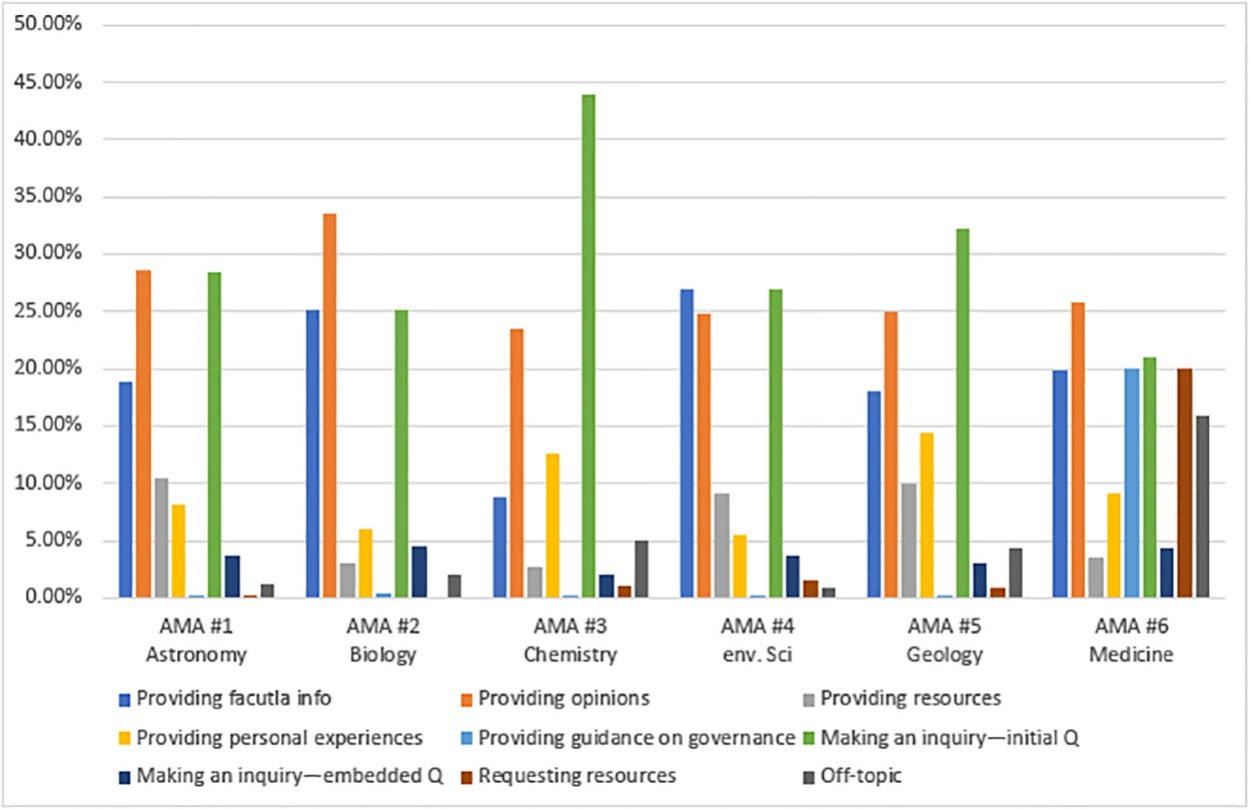
Content features. Note: Percentages were calculated by the number of posts for each code divided by the total number of all the posts coded for CF (i.e., Total #s in the table).

Fig 3 and S6 Table present content features of these AMAs (RQ3c). It is clear that comments on governance were almost non-existent. This may reflect the r/science moderators’ removal of some malicious comments [42]. In addition, AMAs #3 and #5 had slightly more inquiry posts than the rest, while AMA #2 had more posts providing opinions. We observed that the hosts for AMA #2 received many posts that included thanks for the AMA session and that provided positive feedback on their research and the topic of their AMA. Most of the posts making inquiries were presented as initial questions rather than embedded questions. Fig 3 also shows that AMA #2 and #4 had more posts providing factual information than others while AMA #3 had fewer such posts. This may reflect the topics of discussion. The topics of bird genetics (AMA #2) and climate change (AMA #4) may be more familiar to laypeople; nanomaterials (AMA #3) is less likely to be common knowledge among laypeople. Finally, AMA #6 had significantly more off-topic posts than others. This is not necessarily negative. As previous research has indicated [43], off-topic comments may facilitate the well-being of online communities. We also noticed that the hosts for AMA #6 offered participants more opportunities to express their opinions, rather than the straightforward question-answer rapport established in other AMAs.

#### Types of participants

Different types of users participate in r/science AMA conversations. We differentiated between three types of users to identify who was posting comments (RQ3d): the AMA hosts (PID1), participants with a Reddit flair indicating credentials to support opinions (PID2), and participants without a flair (PID3). Table 3 shows that the percentages of hosts’ posts ranged from 13.4% in AMA #3 (Chemistry) to 45.4% in AMA #6 (Medicine). In general, more than half and sometimes over 80% (AMA #3) of the posts were made by participants. This result indicated that participants were actively engaged in these AMAs.

**Table 3:** Number and percentage of contributions by type of user.

	AMA #1 Astronomy	AMA #2 Biology	AMA #3 Chemistry	AMA #4 Env. Sci.	AMA #5 Geology	AMA #6 Medicine
PID1 (host)	63 (27.8%)	70 (34.5%)	31 (13.1%)	70 (28.3%)	41 (20.7%)	114 (45.4%)
PID2 (participant w/ flair)	5 (2.2%)	2 (1.0%)	6 (2.5%)	10 (0.4%)	5 (2.5%)	2 (0.8%)
PID3 (participant wo/ flair)	159 (70.0%)	131 (64.5%)	199 (84.3%)	168 (67.7%)	152 (76.8%)	135 (53.8%)
Total	227	203	236	248	198	251

Note: Percentages were calculated by the number of posts for each code divided by the total number of all the posts coded for PID (i.e., Total in the bottom row).

#### Seeking information posts

**Fig 4:**
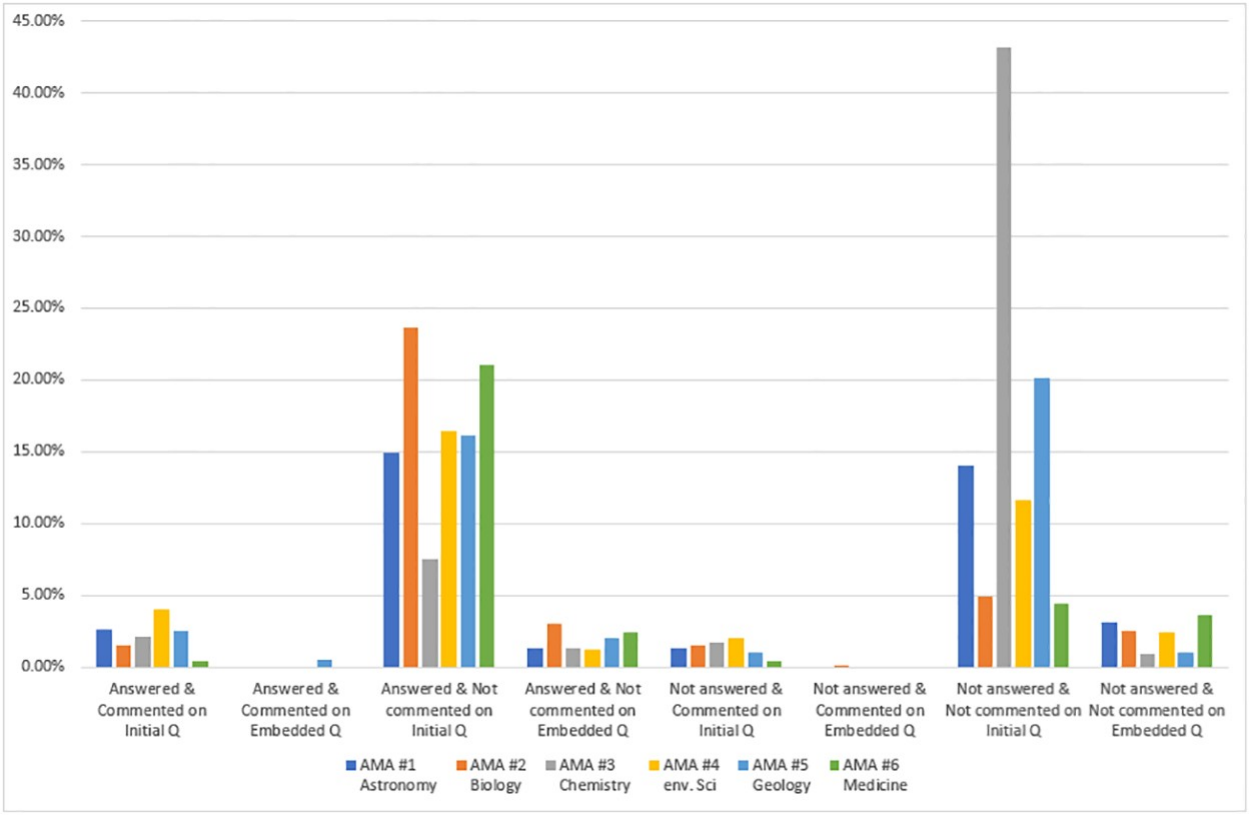
Percentage of posts seeking information based on answer status, comment status, and question location.

We then examined the types of response posts in which AMA participants were seeking information (RQ3e). The code combination of PI1 (seeking information), AS1/AS2 (answered or not), CS1/CS2 (commented on or not), and CF6a/CF6b (initial or embedded question) addresses this question. Regardless of whether posts seeking information were answered or not, they tended to be initial questions that appeared at the start of a discussion thread rather than follow-up questions embedded within the discussion. Furthermore, the posts coded as seeking information tended to receive direct answers rather than comments that did not necessarily reply to the question asked. It makes sense that posts seeking information were initial questions, which were likely asking for information, and that they did not get comments once answered. Some posts did not receive comments or answers; in other words, some questions were left unnoticed. At the same time, the percentages for the results of this code combination (PI1+AS2+CS2+CF6a: seeking information posts that do not get answers or comments and are also initial questions) tend to be lower than the other code combination (PI1+AS1+CS2+CF6a: seeking information posts that get answers but not comments and are initial questions) except for in AMA #3 (Chemistry)—43.2%—and AMA #5 (Geology)—20.2% (see Fig 4). The S7 Table shows the frequency of response types for posts seeking information. This is the nature of AMAs—Reddit users can ask any question, but there is no guarantee that their question will be answered. Further analysis of these questions in the posts may be useful.

#### Seeking discussion posts

**Fig 5:**
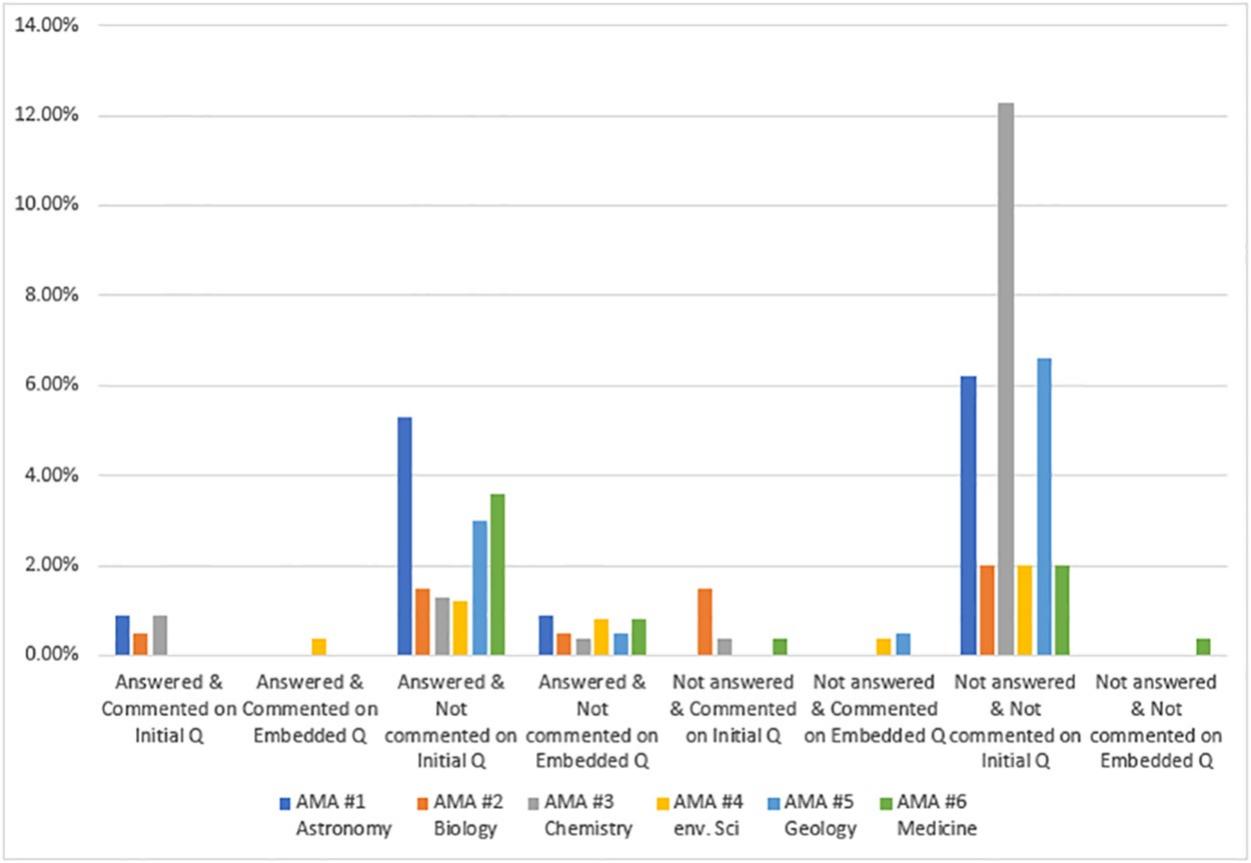
Percentage of posts seeking discussion based on answer status, comment status, and question location.

Next, we examined posts seeking discussion (code: PI2) to see what kinds of responses they received by using the code combination of PI2 (seeking discussion), AS1/AS2 (answered or not), CS1/CS2 (commented on or not), and CF6a/CF6b (initial or embedded questions). The posts that sought discussion were fewer in number than the posts seeking information (see Fig 2), and these posts tended to be initial questions. Whenever these posts received answers, they were not likely to be commented on. Similar to posts seeking information, if these posts were not answered, then they were not likely to receive comments. The latter situation was more prominent in AMA #3 (Chemistry) (see Fig 5 and S8 Table).

## Discussion and conclusions

This examination of participation in an AMA on the r/science subreddit between October 2016 and June 2017 found that scientists who hosted AMA discussions reported overall positive experiences. Most of the hosts who responded to our survey were college faculty with a Ph.D. degree. Over 90% of these hosts work for institutions located in North America or Europe. These scientists appeared to understand the culture of Reddit and how to follow the general rules—either explicitly stated as policies or implicitly implemented by norms. Because their experiences were overwhelmingly positive, it was not surprising that the majority of these scientists responded that they would host r/science AMAs again and that they would recommend hosting r/science AMAs to their colleagues. However, it should be noted that the only responses we received from the survey were either positive or neutral. We suspect that we are not getting the whole picture and that hosts with less positive experiences may have declined to participate. Future research should try to collect data from those who had negative experiences through other sampling strategies, such as snowball sampling.

In terms of the number of questions and responses, there was variation among the range of AMAs studied. Some hosts responded to more questions than others—most often by responding briefly—while those who only responded to a few questions often provided considerably more detailed responses. We are not judging which approach is better, but it would be useful for researchers who host AMAs, and online PES practitioners who assist these researchers, to consider goals or guidelines appropriate to the topic and to decide what approach to take when responding to questions—either responding briefly to as many questions as possible or answering fewer questions but with more in-depth responses. It would also be useful to understand which answering strategies would best meet participants’ expectations, if those expectations were known in advance. Another point for online PES practitioners to consider is that in the AMA format, most answers and comments were focused on initial questions. As such, researchers participating in similar online PES can be advised that they do not need to feel obligated to attempt to answer all of the questions, including embedded questions.

One of the positive experiences that our survey respondents commented on was the collaboration with other researchers as a way to participate in this online PES. Scientists may consider working with their colleagues to host online Q&A sessions including AMAs. In this way, the experience may be less intimidating and burdensome, and more enjoyable. It is also important to emphasize that AMA hosts were not the sole respondents to participants’ questions. Whenever the hosts’ contributions seemed lacking, other participants tended to chime in more. It appears that this openness is intrinsic to the culture of Reddit. This active participation was evident in our analysis of the types of responses. Participants were actively engaged, and were not always content to wait to hear from experts (i.e., the AMA hosts). It is an assurance that, at least in the r/science AMAs that we examined, two-way communication occurred between scientists and the public (i.e., participants). At least some aspects of the potential for online science communication advocated by Brossard [16] and others are realized to some extent in this platform. We speculate that the participants’ active involvement was created by the nature of this environment (i.e., technologically-mediated and inquiry-based) and because of the existing r/science norms. At the same time, online PES practitioners can use r/science AMA as a model to facilitate and participate in similar online PES activities.

In the past, scientists used various means to help lay audiences engage in public participation, such as policy making, dialogue, and knowledge production [4]. Science cafés and science exhibits are used widely, especially in Europe and Asia, for the purpose of creating a dialogue [6]. However, some scientists consider interactions in science cafés ineffective or time-consuming [44]. Online PES is an alternative to face-to-face PES, which has some advantage in its relative ease of access (anyone with an Internet connection can participate) and the possibility for an unlimited number of participants. One of the respondents to our survey commented: “*I could reach hundreds (thousands*?*) of people with very little work*.”

As for limitations, our use of manual coding limited the sample size (six AMAs). However, it should be possible in the future to detect some of the codes automatically, using natural language processing. Second, the survey responses were all positive. This suggests that the AMA hosts who had negative experiences did not respond to our survey. Therefore, the results of the survey should be taken with a grain of salt. Third, we were not able to find general demographic data regarding r/science AMA participants. We are currently conducting another study to address this gap. Fourth, we did not specifically ask about scientists’ prior experiences and motivation with PES. As prior studies suggest, past PES experiences have an impact on future PES activities. Poliakoff and Webb [45] conducted a survey that found past experience with PES is one of the four factors that influences scientists’ intentions to participate in PES again. Other factors include scientists’ positive attitude towards PES, their confidence in PES activities, and their perception of PES being a norm among their colleagues. Our survey also found that recommendations by colleagues was the number one reason why respondents decided to host AMAs in r/science. Future studies should consider asking these types of questions in a survey.

Despite these limitations, this study makes several contributions to investigating this emerging form of public engagement in science. First, we gained an understanding of the attitudes of scientists to participation in r/science AMAs. The survey responses were mostly positive, and they provided first-hand insight into the experience of hosting an AMA in r/science. Second, this study helped uncover the types of interactions that occur in r/science AMAs. Both our in-depth content analysis and the results of our survey will help scientists who are curious about becoming AMA or IAmA hosts (or hosts on similar platforms) to better prepare for the experience. Such preparation could also lead to increased participation, and we hope that more scientists will participate in this emerging form of science communication with the general public. Although r/science recently ceased to conduct their own AMA series, scientists should consider employing other similar venues (e.g. r/AMA, r/IAmA, etc.) for the purpose of science communication. Third, other researchers can apply the coding scheme we developed for other question and answer sites. In short, AMAs hosted via Reddit have the potential to provide unique and interesting opportunities for dialogue between scientists and the general public. In addition, an increased understanding of the AMA process will help scientists interested in using this platform for communicating with the public to better prepare to meet the needs and expectations of participants.

## Supporting information

S1 File Survey instrument for scientists who hosted AMA in science subReddit. (DOCX)

S1 Fig Comparison of poster’s intent to answer a question (PI5) and posts that received answers (AS1). (DOCX)

S1 Table Demographic characteristics of AMA hosts in r/science between October 20, 2016 and June 27, 2017. (DOCX)

S2 Table Codebook for the content analysis. (DOCX)

S3 Table Descriptive statistics of participants and their reported satisfaction with hosting an AMA. (DOCX)

S4 Table Number of posts by AMA hosts and participants containing direct answers to questions (PI5+ PID1/2/3). (DOCX)

S5 Table Poster’s intention. (DOCX)

S6 Table Content Features. (DOCX)

S7 Table Types of responses to posts seeking information. (DOCX)

S8 Table Types of responses to posts seeking discussion. (DOCX)
