# Supplementary material for: An emerging form of public engagement with science: Ask Me Anything (AMA) sessions on Reddit r/science
Source: PLoS One. 2019 May 15;14(5):e0216789. doi: 10.1371/journal.pone.0216789 (PMC6519800; doi:10.1371/journal.pone.0216789)
Supplement: S1 File — (DOCX) [file pone.0216789.s001.docx]

**S1 File. Survey Instrument for Scientists who Hosted AMA in Science SubReddit.**

1. **How did you become involved in hosting an AMA on the “Science” subreddit? Click all that apply.**
2. Colleagues
3. Professional Association
4. Myself
5. Subreddit moderator
6. Students
7. Other
8. **How did you decide on the particular topic?**
9. **How positive or negative was your experience with hosting the AMA?**
10. Very negative
11. Negative
12. Neutral
13. Positive
14. Very positive
15. **Did you receive any assistance from Reddit, such as technical support or assistance from a moderator?**
16. Yes
17. No
18. **What kind of assistance? Click all that apply.**
    1. Technical support—how to use the platform
    2. How to interact with participants
    3. How to prioritize questions
    4. Assistance from a moderator
    5. Other
19. **Was it helpful?**
    1. Yes
    2. No
20. **Why was it not helpful?**
21. **What types of questions did you most enjoy?**
22. **What types of questions did you least enjoy?**
23. **What did you like most about the experience?**
24. **What did you like least about the experience?**
25. **How likely are you willing to host an AMA session again?**
    1. Extremely likely
    2. Somewhat likely
    3. Neither likely nor unlikely
    4. Somewhat unlikely
    5. Extremely unlikely
26. **Have you recommended hosting an AMA to your colleagues?**
    1. Yes
    2. No
27. **How likely are you going to recommend hosting an AMA to your colleagues?**
    1. Extremely likely
    2. Somewhat likely
    3. Neither likely nor unlikely
    4. Somewhat unlikely
    5. Extremely unlikely
28. **What are the lessons you learned from your experience?**
29. **Do you have any suggestions for Reddit regarding hosting an AMA on the “Science” subreddit?**
    1. Yes
    2. No
30. **Please provide specific suggestions:**
31. **What is your gender?**
    1. Male
    2. Female
    3. Prefer not to specify
32. **What is the highest level of school you have completed or the highest degree you have received?**
    1. Doctoral degree
    2. Professional degree (JD, MD)
    3. Master’s degree
    4. Bachelor’s degree in college (4-year)
    5. Other
33. **What is your age?**
    1. 20s
    2. 30s
    3. 40s
    4. 50s
    5. 60s
    6. Above 70
34. **Choose one or more races that you consider yourself to be:**
    1. White
    2. Black or African American
    3. Native American or American Indian
    4. Hispanic or Latino
    5. Asian or Pacific Islander
    6. Other
35. **What best describes your field?**
    1. Astronomy
    2. Biology
    3. Chemistry
    4. Computer Science
    5. Earth Science
    6. Engineering
    7. Geology
    8. Medicine
    9. Nanoscience
    10. Psychology
    11. Physics
    12. Social Science
    13. Other
36. **What best describes the type of organization you work for?**
    1. University or college
    2. Research institution
    3. Government
    4. Not-for-profit organization
    5. Hospital
    6. Private Industry
    7. Other
37. **How long have you been reading the “Science” subreddit?**
    1. Less than a year
    2. 1-3 years
    3. 4-6 years
    4. 7-9 years
    5. More than 10 years
38. **How long have you been actively participating in (e.g., commenting and answering questions) the “Science” subreddit?**
    1. Less than a year
    2. 1-3 years
    3. 4-6 years
    4. 7-9 years
    5. More than 10 years
39. **Would you be willing to participate in a follow-up interview that would take about 30-45 minutes?**
    1. Yes
    2. No
40. **If yes, please provide your contact information below:**
