# Supplementary material for: An emerging form of public engagement with science: Ask Me Anything (AMA) sessions on Reddit r/science
Source: PLoS One. 2019 May 15;14(5):e0216789. doi: 10.1371/journal.pone.0216789 (PMC6519800; doi:10.1371/journal.pone.0216789)
Supplement: S1 Fig — (DOCX) [file pone.0216789.s002.docx]

**S1 Figure. Comparison of Poster’s Intent to Answer a Question (PI5) and Posts that Received Answers (AS1).**
