# Supplementary material for: An emerging form of public engagement with science: Ask Me Anything (AMA) sessions on Reddit r/science
Source: PLoS One. 2019 May 15;14(5):e0216789. doi: 10.1371/journal.pone.0216789 (PMC6519800; doi:10.1371/journal.pone.0216789)
Supplement: S1 Table — (DOCX) [file pone.0216789.s003.docx]

**S1 Table. Demographic Characteristics of AMA Hosts in r/science**

**between October 20, 2016 and June 27, 2017.**

| Gender | Female | 123 (39.0%) |
| --- | --- | --- |
|  | Male | 192 (61.0%) |
|  | *Total* | *315* |
| Terminal degree | Yes | 222 (70.5%) |
|  | No | 93 (29.5%)* |
|  | *Total* | *315* |
| Type of terminal degree | Ph.D. | 204 (91.9%) |
|  | M.D. | 11 (5.0%) |
|  | Ph.D. and M.D. | 6 (2.7%) |
|  | Ph.D. and D.V.M. | 1 (0.5%) |
|  |  | 222 |
| Country of employment |  |  |
|  | Australia | 4 (1.3%) |
|  | Austria | 1 (0.3%) |
|  | Canada | 7 (2.2%) |
|  | England | 26 (8.3%) |
|  | France | 1 (0.3%) |
|  | Germany | 5 (1.6%) |
|  | India | 1 (0.3%) |
|  | Israel | 1 (0.3%) |
|  | Italy | 1 (0.3%) |
|  | Netherlands | 5 (1.6%) |
|  | New Zealand | 1 (0.3%) |
|  | Norway | 2 (0.6%) |
|  | Scotland | 2 (0.6%) |
|  | South Africa | 1 (0.3%) |
|  | Spain | 1 (0.3%) |
|  | Sweden | 1 (0.3%) |
|  | Switzerland | 3 (1.0%) |
|  | United States | 252 (80.0%) |
|  | *Total* | *315* |
| AMA category |  |  |
|  | Animal Science | 3 (1.0%) |
|  | Astronomy | 10 (3.2%) |
|  | Biology | 46 (14.6%) |
|  | Cancer | 4 (1.3%) |
|  | Chemistry | 33 (10.5%) |
|  | Computer Science | 4 (1.3%) |
|  | Engineering | 21 (6.7%) |
|  | Environment | 45 (14.3%) |
|  | Epidemiology | 3 (1.0%) |
|  | Geology | 26 (8.3%) |
|  | Health | 5 (1.6%) |
|  | Medicine | 48 (15.2%) |
|  | N/A | 10 (3.2%) |
|  | Nanoscience | 5 (1.6%) |
|  | Neuroscience | 19 (6.0%) |
|  | Paleontology | 2 (0.6%) |
|  | Physics | 5 (1.6%) |
|  | Psychology | 14 (4.4%) |
|  | Social Science | 12 (3.8%) |
|  | *Total* | *315* |

Note: *”No” includes those whom we were not able to identify.
