# Supplementary material for: An emerging form of public engagement with science: Ask Me Anything (AMA) sessions on Reddit r/science
Source: PLoS One. 2019 May 15;14(5):e0216789. doi: 10.1371/journal.pone.0216789 (PMC6519800; doi:10.1371/journal.pone.0216789)
Supplement: S2 Table — (DOCX) [file pone.0216789.s004.docx]

**S2 Table. Codebook for the Content Analysis.**

| **Top-level categories** | **Coding system** | **Justifications of final coding system (“Apply this code if”)** |
| --- | --- | --- |
| Poster’s intensions | PI1. Seeking information | Query is a “vertical question,” a question to which a factual answer exists or a question seeking an expert (AMA host) opinion; a fact is defined here as something that could be looked up in a reference source; includes questions that were meant to be rhetorical but were then answered |
|  | PI2. Seeking discussion | Query is a “horizontal question,” a question that generates discussion rather than a single response; posts are coded in this category if questioner is asking for others’ opinions rather than a factual answer because these questions will likely invoke discussion; includes questions that were meant to be rhetorical but were then answered |
|  | PI3. Non-questions, comments | Post includes comments that do not include a question; also includes rhetorical questions that are not answered |
|  | PI4. Furthering discussion, interaction among users | Poster answers/responds to information presented in the AMA in order to contribute to the discussion; “Furthering discussion” includes asking additional questions or providing additional facts, opinions, or comments like “That’s a good idea for further research”; PI4. applies to *any* post after the first post in a thread that contributes to the discussion in that thread |
|  | PI5. Answering a question | Any post that directly answers a question, regardless of the quality of the answer; answer can include linking to content hosted elsewhere; this does not include responses that offer follow-up questions or agreement with the original question like “I would also like to know this as well!” |
| Answer status | AS1. Answered | Post contains one or more questions that receive a direct answer (regardless of the quality of the answer) by either the AMA host or another poster; this answer does not need to be the post immediately after the question; a post should be coded as AS1 if even one of the questions asked was answered, no matter how many questions the poster asked; any post that receives AS1 cannot also be coded AS2 |
|  | AS2. Not answered | Post contains one or more questions that do not receive a direct answer by either by the AMA host or another poster; any post that receives AS2 cannot also be coded AS1 |
| Comment status | CS1. Commented on | Post contains one or more questions that receive a comment but not a direct answer, e.g. a follow-up question or agreement with the original question like “I would also like to know this as well!”; any post that receives CS1 cannot also be coded CS2 |
|  | CS2: Not commented on | Post contains one or more questions that receive no comment; any post that receives CS2 cannot also be coded CS1 |
| Poster’s identity | PID1. Host | Poster is tagged as AMA host |
|  | PID2. Participant – flair | Poster is tagged with vetted flair |
|  | PID3. Participant – no flair | Post has no tags |
| Content features | CF1. Providing factual information | Post (either a question or response) contains information based on facts, i.e., something that could be looked up in a reference source; this category includes AMA hosts’ own work and conclusions, the conclusions and work of other scholars, and factual information provided by AMA participants; category includes any posts that are presented as fact rather than the poster’s opinion; does not include references to personal experience |
|  | CF2. Providing opinions | Post by either the AMA host or other participants that relays the poster’s opinions rather than factual information (e.g. “We think…”, “I believe…”, “We hypothesize…”, “I am interested in…”); post provides a general sense of the poster’s feelings about or reception of the content |
|  | CF3. Providing resources | Post shares direct citations or links to resources, the AMA host’s own work, or work of other researchers; the resources provided can refer to other researchers, their work, famous concepts or theories, established theoretical frameworks, media publications, news stories, or similar; these resources are linked directly from the post, or the poster provides enough information that the reader will be able to access the resource (e.g., citation, URL) |
|  | CF4. Providing personal experience | Post contains explicit or implicit references to participant’s personal information such as education, work experience, area of expertise or lack of expertise, life experience, or other types of personal information; post is focused on the personal aspect of someone’s experience |
|  | CF5. Providing guidance on forum governance | Post contains explanation of the forum rules and procedures |
|  | CF6a. Making an inquiry – initial question | Post contains a question that begins a thread and seeks a response; does not include rhetorical questions; if a post is coded CF6a, it must also be coded with either PI1 or PI2, and one of the QS codes |
|  | CF6b. Making an inquiry – embedded question | Post contains a question that is embedded within a thread and seeks a response; does not include rhetorical questions; if a post is coded CF6b, it must also be coded with either PI1 or PI2, and one of the QS codes |
|  | CF7. Requesting resources | Poster requests that the AMA host or other participants suggest outside resources for further reading or viewing (e.g., “Is there a book you’d recommend for someone interested in satellite communication?”) |
|  | CF8. Off-topic comment | Post includes content that does not relate to the topic of the AMA (e.g., “I imagine you spend a lot of time in cold places due to the nature of your work . . . what is your go to crock pot recipe?”, "What kind of pizza do you like?"); posts that receive this code may also include on-topic content |
