# Supplementary material for: An emerging form of public engagement with science: Ask Me Anything (AMA) sessions on Reddit r/science
Source: PLoS One. 2019 May 15;14(5):e0216789. doi: 10.1371/journal.pone.0216789 (PMC6519800; doi:10.1371/journal.pone.0216789)
Supplement: S3 Table — (DOCX) [file pone.0216789.s005.docx]

**S3 Table. Descriptive Statistics of Participants and their Reported Satisfaction with Hosting an AMA.**

| ****How to become involved in AMA?*** | ***N*** |
| --- | --- |
| Colleagues | 27 |
| Professional Assoc. | 16 |
| Myself | 14 |
| Moderator | 8 |
| Students | 2 |
| Other | 16 |
| *Total* | *83* |
| ***Gender*** |  |
| Male | 36 |
| Female | 32 |
| Prefer not to specify | 2 |
| *Total* | *70* |
| ***Degree*** |  |
| Doctoral degree | 52 |
| Professional degree (JD, MD) | 1 |
| Master’s degree | 10 |
| Bachelor’s degree (4-year) | 7 |
| *Total* | *70* |
| ***Age*** |  |
| 20s | 13 |
| 30s | 30 |
| 40s | 9 |
| 50s | 9 |
| 60s | 7 |
| Above 70 | 2 |
| *Total* | *70* |
| ****Race*** |  |
| White | 66 |
| Black or African American | 0 |
| Native American or American Indian | 0 |
| Hispanic or Latino | 1 |
| Asian or Pacific Islander | 3 |
| Other | 3 |
| *Total* | *73* |
| ***Disciplines*** |  |
| Astronomy | 1 |
| Biology | 18 |
| Chemistry | 7 |
| Computer Science | 2 |
| Earth Science | 7 |
| Engineering | 4 |
| Geology | 2 |
| Medicine | 4 |
| Psychology | 9 |
| Physics | 3 |
| Social Science | 4 |
| Other | 9 |
| *Total* | *70* |
| ***Organizations*** |  |
| University or college | 50 |
| Research institution | 4 |
| Government | 6 |
| Not-for-profit organization | 4 |
| Private industry | 3 |
| Other | 3 |
| *Total* | *70* |
| *****Years of reading r/science subreddit*** |  |
| Less than a year | 48 |
| 1-3 years | 14 |
| 4-6 years | 5 |
| 7-9 years | 2 |
| *Total* | *69* |
| ***Years of participating r/science subreddit*** |  |
| Less than a year | 56 |
| 1-3 years | 9 |
| 4-6 years | 5 |
| *Total* | *70* |
| ***Experience with AMA*** |  |
| Neutral | 4 |
| Positive | 32 |
| Very positive | 34 |
| *Total* | *70* |
| ***How likely to host AMA again*** |  |
| Extremely likely | 41 |
| Somewhat likely | 25 |
| Neither likely nor unlikely | 2 |
| Somewhat unlikely | 2 |
| *Total* | *70* |
| ***Recommended AMA*** |  |
| Yes | 59 |
| No | 11 |
| *Total* | *70* |
| ***Likely recommend AMA#*** |  |
| Somewhat likely | 5 |
| Neither likely nor unlikely | 4 |
| Somewhat unlikely | 2 |
| *Total* | *11* |
| ***Received assistance*** |  |
| Yes | 42 |
| No | 28 |
| ***Kinds of assistance received**** |  |
| Technical support | 25 |
| How to interact with participants | 15 |
| How to prioritize questions | 15 |
| Assistance from a moderator | 16 |
| Other | 3 |
| *Total* | *74* |
| ***Was the assistance helpful? ##*** |  |
| Yes | 41 |
| No | 1 |
| *Total* | *42* |
|  |  |

Note: The questions marked with * allowed multiple answers. The question marked with ** was not answered by one respondent. The question marked # was asked only for those who responded to “NO” to the previous question: “have you recommended AMA to colleagues?” The question marked ## was asked only for those who responded to “Yes” to the previous question of receiving assistance.
