# Supplementary material for: An emerging form of public engagement with science: Ask Me Anything (AMA) sessions on Reddit r/science
Source: PLoS One. 2019 May 15;14(5):e0216789. doi: 10.1371/journal.pone.0216789 (PMC6519800; doi:10.1371/journal.pone.0216789)
Supplement: S4 Table — (DOCX) [file pone.0216789.s006.docx]

**S4 Table. Number of Posts by AMA Hosts and Participants Containing Direct Answers to Questions (PI5+ PID1/2/3).**

|  | AMA #1  Astronomy | AMA #2  Biology | AMA #3  Chemistry | AMA #4  Env. Sci. | AMA #5  Geology | AMA #6  Medicine |
| --- | --- | --- | --- | --- | --- | --- |
| PI5 + PID 1 | 57  (25.1%) | 64 (31.5%) | 28  (11.9%) | 68 (27.4%) | 38  (19.2%) | 104 (41.4%) |
| PI5 + PID 2 | 2 (0.9%) | 0 | 1 (0.4%) | 0 | 0 | 0 |
| PI5 + PID 3 | 8 (3.5%) | 6 (3.0%) | 3 (1.3%) | 22 (8.9%) | 20 (10.1%) | 7 (2.8%) |

Note: Percentages were calculated by the number of posts coded as PI5 + PID1/PID2/PID3 divided by the total number of posts.
