# Supplementary material for: An emerging form of public engagement with science: Ask Me Anything (AMA) sessions on Reddit r/science
Source: PLoS One. 2019 May 15;14(5):e0216789. doi: 10.1371/journal.pone.0216789 (PMC6519800; doi:10.1371/journal.pone.0216789)
Supplement: S5 Table — (DOCX) [file pone.0216789.s007.docx]

**S5 Table. Poster’s Intention.**

|  | AMA #1  Astronomy | AMA #2  Biology | AMA #3  Chemistry | AMA #4  Env. Sci. | AMA #5  Geology | AMA #6  Medicine |
| --- | --- | --- | --- | --- | --- | --- |
| **PI1: Seeking Info** | 85 (23.4%) | 76 (22.8%) | 135 (42.9%) | 94 (23.3%) | 87 (29.0%) | 80 (19.7%) |
| **PI2: Seeking discussion** | 30 (8.2%) | 12 (3.6%) | 36 (11.4%) | 12 (3.0%) | 21 (7.0%) | 28 (6.9%) |
| **PI3: Non-question** | 122 (33.5%) | 119 (35.6%) | 72 (22.9%) | 143 (35.5%) | 94 (31.3%) | 146 (35.9%) |
| **PI4: Furthering**  **Discussion** | 60 (16.5%) | 58 (17.4%) | 40 (12.7%) | 64 (15.9%) | 40 (13.3%) | 42 (10.3%) |
| **PI5: Answering a question** | 67 (18.4%) | 69 (20.7%) | 32 (10.2%) | 90 (22.3%) | 58 (19.3%) | 111 (27.3%) |
| **Total** | 364 | 334 | 315 | 403 | 300 | 407 |

Note: Percentages were calculated by the number of posts for each code divided by the total number of all the posts coded for PI (i.e., Total #s in the table).
