# Supplementary material for: An emerging form of public engagement with science: Ask Me Anything (AMA) sessions on Reddit r/science
Source: PLoS One. 2019 May 15;14(5):e0216789. doi: 10.1371/journal.pone.0216789 (PMC6519800; doi:10.1371/journal.pone.0216789)
Supplement: S6 Table — (DOCX) [file pone.0216789.s008.docx]

**S6 Table. Content Features.**

|  | AMA #1  Astronomy | AMA #2  Biology | AMA #3  Chemistry | AMA #4  Env. Sci. | AMA #5  Geology | AMA #6  Medicine |
| --- | --- | --- | --- | --- | --- | --- |
| **CF1: Providing factual information** | 62 (18.9%) | 71 (25.1%) | 32  (8.9%) | 92 (27.0%) | 56  (17.3%) | 83  (19.8%) |
| **CF2: Providing opinions** | 94  (28.7%) | 95  (33.6%) | 84  (23.5%) | 85 (24.9%) | 79  (24.4%) | 108  (25.8%) |
| **CF3: Providing resources** | 34  (10.4%) | 9  (3.1%) | 10  (2.8%) | 31 (9.1%) | 27  (8.3%) | 15  (3.6%) |
| **CF4: Providing personal experiences** | 27  (8.2%) | 17  (6.0%) | 45  (12.6%) | 19 (5.6%) | 40  (12.3%) | 38  (9.1%) |
| **CF5: Providing guidance on governance** | 1 (0.3%) | 1 (0.4%) | 1 (0.3%) | 1 (0.3%) | 1 (0.3%) | 1 (0.2%) |
| **CF6a: Making an inquiry—initial q** | 93  (28.4%) | 71  (25.1%) | 157  (43.9%) | 92 (27.0%) | 94  (29.0%) | 88  (21.0%) |
| **CF6b: Making an inquiry—embedded q** | 12 (3.7%) | 13 (4.6%) | 7 (2.0%) | 13 (3.8%) | 10 (3.1%) | 18 (4.3%) |
| **CF7: Requesting resources** | 1 (0.3%) | 0 | 4 (1.1%) | 5 (1.5%) | 3 (0.9%) | 1 (0.2%) |
| **CF8: Off-topic** | 4 (1.2%) | 6 (2.1%) | 18 (5.0%) | 3 (0.9%) | 14 (4.3%) | 67 (16.0%) |
| **Total** | 328 | 283 | 358 | 341 | 324 | 419 |

Note: Percentages were calculated by the number of posts for each code divided by the total number of all the posts coded for CF (i.e., Total #s in the table)
