# Supplementary material for: An emerging form of public engagement with science: Ask Me Anything (AMA) sessions on Reddit r/science
Source: PLoS One. 2019 May 15;14(5):e0216789. doi: 10.1371/journal.pone.0216789 (PMC6519800; doi:10.1371/journal.pone.0216789)
Supplement: S7 Table — (DOCX) [file pone.0216789.s009.docx]

**S7 Table. Types of Responses to Posts Seeking Information.**

|  | AMA #1  Astronomy | AMA #2  Biology | AMA #3  Chemistry | AMA #4  Env. Sci. | AMA #5  Geology | AMA #6  Medicine |
| --- | --- | --- | --- | --- | --- | --- |
| PI1 + AS1 + CS1 + CF6a | 6 (2.6%) | 3 (1.5%) | 5 (2.1%) | 10 (4.0%) | 5 (2.5%) | 1 (0.4%) |
| PI1 + AS1 + CS1 + CF6b | 0 | 0 | 0 | 0 | 1 (0.5%) | 0 |
| PI1 + AS1 + CS2 + CF6a | 34 (15.0%) | 48 (23.6  %) | 18  (7.6%) | 41 (16.5%) | 32 (16.2%) | 53 (21.1%) |
| PI1 + AS1 + CS2 + CF6b | 3 (1.3%) | 6 (3.0%) | 3 (1.3%) | 3 (1.2%) | 4 (2.0%) | 6 (2.4%) |
| PI1 + AS2 + CS1 + CF6a | 3 (1.3%) | 3 (1.5%) | 4 (1.7%) | 5 (2.0%) | 2 (1.0%) | 1 (0.4%) |
| PI1 + AS2 + CS1 + CF6b | 0 | 1 (0.5%) | 0 | 0 | 0 | 0 |
| PI1 + AS2 + CS2 + CF6a | 32 (14.1%) | 10 (4.9%) | 102  (43.2%) | 29 (11.7%) | 40 (20.2%) | 11 (4.4%) |
| PI1 + AS2 + CS2 + CF6b | 7 (3.1%) | 5 (2.4%) | 2 (0.8%) | 6 (2.4%) | 2 (1.0%) | 9 (3.6%) |

Note: PI1 (information seeking); AS1 (answered); AS2 (not answered); CS1 (commented on); CS2 (not commented on); CF6a (initial question); CF6a (embedded question). Percentages were calculated by the number of posts in each category divided by the total number of posts.
