# Supplementary material for: An emerging form of public engagement with science: Ask Me Anything (AMA) sessions on Reddit r/science
Source: PLoS One. 2019 May 15;14(5):e0216789. doi: 10.1371/journal.pone.0216789 (PMC6519800; doi:10.1371/journal.pone.0216789)
Supplement: S8 Table — (DOCX) [file pone.0216789.s010.docx]

**S8 Table. Types of Responses to Posts Seeking Discussion.**

|  | AMA #1  Astronomy | AMA #2  Biology | AMA #3  Chemistry | AMA #4  Env. Sci. | AMA #5  Geology | AMA #6  Medicine |
| --- | --- | --- | --- | --- | --- | --- |
| PI2 + AS1 + CS1 + CF6a | 2 (0.9%) | 1 (0.5%) | 2 (0.8%) | 0 | 0 | 0 |
| PI2 + AS1 + CS1 + CF6b | 0 | 0 | 0 | 1 (0.4%) | 0 | 0 |
| PI2 + AS1 + CS2 + CF6a | 12 (5.3%) | 3 (1.5%) | 3 (1.3%) | 3 (1.2%) | 6 (3.0%) | 19 (7.6%) |
| PI2 + AS1 + CS2 + CF6b | 2 (0.9%) | 1 (0.5%) | 1 (0.4%) | 2 (0.8%) | 1 (0.5%) | 2 (0.8%) |
| PI2 + AS2 + CS1 + CF6a | 0 | 3 (1.5%) | 1 (0.4%) | 0 | 0 | 1 (0.4%) |
| PI2 + AS2 + CS1 + CF6b | 0 | 0 | 0 | 1 (0.4%) | 1 (0.5%) | 0 |
| PI2 + AS2 + CS2 + CF6a | 14 (6.2%) | 4 (2.0%) | 29 (12.3%) | 5 (2.0%) | 13 (6.6%) | 5 (2.0%) |
| PI2 + AS2 + CS2 + CF6b | 0 | 0 | 0 | 0 | 0 | 1 (0.4%) |

Note: PI2 (discussion seeking); AS1 (answered); AS2 (not answered); CS1 (commented on); CS2 (not commented on); CF6a (initial question); CF6a (embedded question). Percentages were calculated by the number of posts in each category divided by the total number of posts.
